# A Legacy to the Metropolitan Asylums Board

**Published:** 1913-03-01

**Authors:** 


### THE EDITOR'S LETTER-BOX.

### "A Legacy to the Metropolitan Asylums Board."

*To the Editor of* The Hospital.

Sir,—Referring to the article under the above heading in your issue of February 15, would you kindly permit me to give expression to a suggestion for the utilisation of the money in question, which I consider would be perfectly equitable, and would, moreover, obviate the questionable act of diverting rightfully acquired funds when such a course would apparently be in direct contravention of the donor's wishes?

As one having some knowledge of the M.A.B., and of how insistent they are in obtaining full value for every penny spent, and also having considerable interest in the children of their excellently managed institutions, I have been much concerned with the difficulties which have sprung up, owing to lack of funds which could be directly applied without incurring risk of surcharge by the L.G.B. auditor, when it has been desired to start a boy out in life, to apprentice him to a trade, to pay his passage abroad, etc.

In my experience there is no lack of good openings for girls properly trained for domestic service, but in the case of boys who have been treated for ringworm of the scalp or ophthalmia the difficulties of securing a start for them in anything but blind-alley occupations are enormous; and it is, in my opinion, in this way that the money would be most usefully employed, while the managers at the same time would be faithfully observing the deliberately expressed wishes of the generous donor.—Your obedient servant, One Who Knows.

